# Eosinophilic Gastritis in a Patient Previously Treated with Dupilumab

**DOI:** 10.1155/2020/6381670

**Published:** 2020-06-03

**Authors:** Masaya Iwamuro, Toshi Murakami, Takehiro Tanaka, Shohei Oka, Seiji Kawano, Yoshiro Kawahara, Hiroyuki Okada

**Affiliations:** ^1^Department of Gastroenterology and Hepatology, Okayama University Graduate School of Medicine, Dentistry and Pharmaceutical Sciences, Okayama 700-8558, Japan; ^2^Satsuki Naika Clinic, Okayama 703-8258, Japan; ^3^Department of Pathology, Okayama University Graduate School of Medicine, Dentistry and Pharmaceutical Sciences, Okayama 700-8558, Japan; ^4^Department of Practical Gastrointestinal Endoscopy, Okayama University Hospital, Okayama 700-8558, Japan

## Abstract

A 77-year-old Japanese man with bronchial asthma was treated with dupilumab. Dupilumab treatment was discontinued at the patient's request after two injections separated by a 2-week interval. The blood eosinophil count was elevated, and an esophagogastroduodenoscopy performed 3 months after dupilumab treatment revealed gastric ulcers; subsequently, eosinophilic gastritis was diagnosed from biopsy examinations. The gastric lesions were resolved by steroid administration. This case report underscores that eosinophil-associated gastrointestinal diseases should be considered in the differential diagnosis of gastric lesions occurring in patients who were treated with dupilumab.

## 1. Introduction

Dupilumab, a human monoclonal antibody against interleukin-4 (IL-4) and IL-13, is a subcutaneous injectable prescription medicine used for the treatment of asthma and atopic dermatitis [1, 2]. IL-4 and IL-13 initiate type 2 helper T-cell (Th2) mediated inflammatory processes, such as immunoglobulin *E* (IgE) production, smooth muscle contraction, mucus production, and innate cell recruitment to sites of inflammation, leading to allergic diseases [3]. As dupilumab inhibits both IL-4 and IL-13 signaling, it has beneficial effects in patients with asthma and atopic dermatitis. The efficacy, safety, and tolerability of dupilumab have been investigated and confirmed in clinical trials [4, 5]. However, a higher frequency of eosinophilia in dupilumab-treated patients has been reported both in clinical studies [6, 7] and a real-world study [8].

Herein, we have reported the case of a patient with asthma who developed eosinophilic gastritis after the administration of dupilumab, with a focus on the possible pathogenesis of eosinophil infiltration in the stomach.

## 2. Case Presentation

A 77-year-old Japanese man had visited a hospital with respiratory distress, cough, and sputum. The patient had hyperlipidemia, hypertension, atrophic rhinitis, duodenal ulcers, hyperuricemia, prostatic hypertrophy, and overactive bladder, for which he had been taking pravastatin, cilnidipine, trichlormethiazide, lansoprazole, febuxostat, silodosin, mirabegron, and a probiotic *Lactobacillus* preparation. He was a social drinker and an ex-smoker who smoked 30 cigarettes/day for 13 years. He had received *Helicobacter pylori* eradication treatment at 68 years of age. Although the patient had a history of paranasal sinusitis and occasionally suffered from a cough, he had not previously been diagnosed with bronchial asthma.

His respiratory distress, cough, and sputum were improved by the temporary administration of formoterol inhaler and antibiotics. However, his symptoms reemerged after 3 months, and he visited a clinic. At that time, lung auscultation revealed a wheeze in both the lungs. His oxygen saturation was 89%–91%, his forced expiratory volume percentage in one second was 76.9%, his peak expiratory flow rate was 22.5%, and his exhaled nitric oxide level was 29–31 ppb. The laboratory tests showed increased values for C-reactive protein (1.53 mg/dL) and immunoglobulin *E* (740 IU/mL), whereas the number of white blood cells was within the normal range (6,900 *μ*L). Radioallergosorbent tests were broadly positive for multiple allergens, such as house dust, mites, pollens, candida, and wheat. Fluticasone, formoterol, montelukast, and azithromycin were administered in addition to intravenous methylprednisolone. Although his medication was changed to vilanterol, fluticasone, and montelukast, his respiratory distress, cough, and sputum production were not completely resolved. Thus, dupilumab was administered twice separated by a 2-week interval. However, dupilumab treatment was discontinued at the patient's request because his symptoms were unchanged. Although his eosinophil count was within the normal range before the administration of dupilumab, it varied from 152 to 860 *μ*L after dupilumab injections were initiated. The patient was subsequently treated with montelukast, bilastine, and zinc acetate.

**Figure 1:**
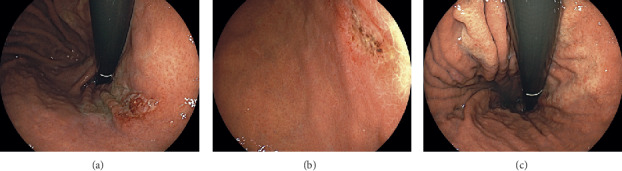
Esophagogastroduodenoscopy images. Esophagogastroduodenoscopy performed 3 months after dupilumab treatment revealed gastric ulcers in the lesser curvature of the cardia (a) and posterior wall of the gastric body (b). Whitish mucosa was also noted in the lesser curvature of the gastric body (c).

**Figure 2:**
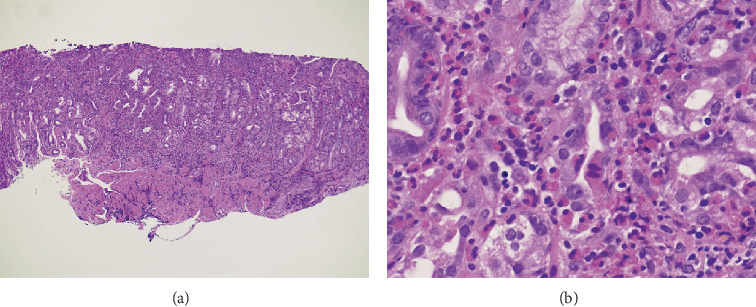
Pathology images. Biopsy from the gastric ulcers showed the infiltration of eosinophils (a × 4; b × 40).

**Figure 3:**
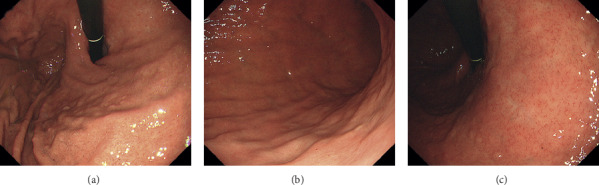
Esophagogastroduodenoscopy images. Endoscopy performed 4 weeks after prednisone administration revealed the resolution of gastric ulcers. No eosinophils were identified in the biopsied specimens.

The patient underwent an esophagogastroduodenoscopy 3 months after the dupilumab treatment for annual screening purposes. Esophagogastroduodenoscopy revealed gastric ulcers in the lesser curvature of the cardia (Figure 1(a)) and in the posterior wall of the gastric body (Figure 1(b)). Whitish mucosa was also observed in the lesser curvature of the gastric body (Figure 1(c)). Although the patient had undergone annual esophagogastroduodenoscopy for 9 years, gastric ulcers or whitish mucosa had not previously been identified. Biopsy of the gastric ulcers revealed more than 100 eosinophils per high-power field, which were infiltrating into the lamina propria and even the epithelium of the gastric mucosa (Figure 2); no neoplastic cells were noted. Consequently, a diagnosis of eosinophilic gastritis was made. The laboratory tests showed slightly increased values for C-reactive protein (0.33 mg/dL) and immunoglobulin *E* (366 IU/mL), whereas white blood cells (6,900 *μ*L) and eosinophils (5.3%) were within the normal ranges. Computed tomography scanning showed no remarkable changes in the gastrointestinal tract. Consequently, 30 mg prednisone was administered to treat eosinophilic gastritis. Esophagogastroduodenoscopy performed 4 weeks after prednisone administration revealed the resolution of gastric ulcers (Figure 3). No eosinophils were identified in the biopsied specimens.

## 3. Discussion

Several randomized, placebo-controlled phase 3 trials revealed that dupilumab was effective for moderate-to-severe atopic dermatitis [9–11]. Alexis et al. performed post hoc analysis from three phase 3 trials assessing the efficacy and safety of dupilumab vs placebo and identified that serious adverse events occurred more frequently in the placebo groups [11]. However, a real-life study in a French multicenter adult cohort revealed a higher frequency of eosinophilia and conjunctivitis in patients with atopic dermatitis treated with dupilumab, compared with clinical trials [12].

Placebo-controlled phase 3 trials showed the efficacy of dupilumab for patients with moderate-to-severe or severe asthma [6, 7, 13]. In one phase 3 trial involving 1,902 patients with uncontrolled asthma [7], 52/1,264 patients in the dupilumab-treated group (4.1%) had eosinophilia, which was associated with symptoms such as the deterioration of chronic eosinophilic pneumonia and hypereosinophilia in four patients. Meanwhile, eosinophilia was observed in 4/638 patients in the placebo group (0.6%). Another phase 3 trial showed that transient blood eosinophilia was more frequently observed in the dupilumab group (14/103 patients, 13.6%) than in the placebo group (1/107 patients, 0.9%). These results indicated that dupilumab treatment may increase the blood eosinophil count in some patients with atopic dermatitis and/or asthma. As dupilumab is considered to inhibit the migration of eosinophils into tissues by blocking IL-4 and IL-13 signaling, it may transiently increase circulating blood eosinophil counts [7, 14]. Another possible mechanism of eosinophilia observed in clinical trials was the withdrawal of glucocorticoids in dupilumab-treated patients, which caused the elevation of blood eosinophils [6, 7].

Other adverse events reported in association with dupilumab included nasopharyngitis, bronchitis, sinusitis [15], injection-site reactions [6], headache [16], skin infections, cutaneous psoriatic lesions [17], alopecia [18, 19], and chronic eosinophilic pneumonia [20]. To the best of our knowledge, the development of gastrointestinal diseases in association with dupilumab use has not been reported previously. Thus, this report is the first to describe a patient with eosinophilic gastritis that occurred after the use of dupilumab.

In the present patient, dupilumab was administered twice separated by a 2-week interval for the treatment of asthma, and dupilumab treatment was discontinued thereafter. Esophagogastroduodenoscopy performed 3 months after dupilumab treatment revealed eosinophilic gastritis. There are several hypotheses regarding the occurrence of eosinophil infiltration in the present patient's stomach. First, the migration of eosinophil into organs was blocked by dupilumab administration, and the chemotaxis behavior may be altered when the migration was resumed after the withdrawal of dupilumab, resulting in stomach-predominant infiltration of eosinophils. Second, the stomach may escape the chemotaxis-sparing effect of dupilumab, and eosinophils converged in the gastric mucosa. Third, the patient was hyperallergic, which caused both asthma and eosinophilic gastritis. Eosinophilic gastroenteritis is frequently found in patients with atopic conditions [21]. Therefore, eosinophilic gastritis might occur coincidentally, independent of dupilumab treatment. However, these hypotheses are speculative and not substantiated by any supporting facts. Because this report includes only one patient with such a case, further investigation is required to clarify the relationship between eosinophilic gastritis and dupilumab use.

Menzella et al. reported a patient with chronic eosinophilic pneumonia that developed after the tenth injection of dupilumab [20]. Although a great improvement in asthma control was obtained, the patient manifested eosinophilia, fever, and bilateral pulmonary thickening. The authors described a progressive increase in blood eosinophil count after dupilumab injections were started, followed by the onset of chronic eosinophilic pneumonia. In the present patient, blood eosinophil count was also elevated after the administration of dupilumab. These results suggested that end-organ manifestations of eosinophilia may occur, regardless of whether the patient is receiving dupilumab treatment or treatment has been discontinued. It was also noteworthy that the patient had no gastrointestinal symptoms despite having multiple stomach ulcers secondary to eosinophil infiltration. Although screening esophagogastroduodenoscopy after administration of dupilumab may reveal asymptomatic gastrointestinal involvement—particularly in patients with eosinophilia—this concept needs further investigation. We hope this report will raise awareness of the potential for eosinophil-associated gastrointestinal disease following dupilumab use.

In conclusion, we encountered a patient with asthma who developed eosinophilic gastritis 3 months after dupilumab use. This case supports the consideration of eosinophilic gastritis in the differential diagnosis of gastric lesions emerging in patients who were treated with dupilumab.

## References

[1] Rathinam K. K., Abraham J. J., Vijayakumar T. M. (2019). Dupilumab in the treatment of moderate to severe asthma: an evidence-based review. *Current Therapeutic Research*.

[2] Kraft M., Worm M. (2017). Dupilumab in the treatment of moderate-to-severe atopic dermatitis. *Expert Review of Clinical Immunology*.

[3] Bao K., Reinhardt R. L. (2015). The differential expression of IL-4 and IL-13 and its impact on type-2 immunity. *Cytokine*.

[4] de Bruin-Weller M., Thaçi D., Smith C. H. (2018). Dupilumab with concomitant topical corticosteroid treatment in adults with atopic dermatitis with an inadequate response or intolerance to ciclosporin a or when this treatment is medically inadvisable: a placebo-controlled, randomized phase III clinical trial (LIBERTY AD CAFÉ). *British Journal of Dermatology*.

[5] Blauvelt A., de Bruin-Weller M., Gooderham M. (2017). Long-term management of moderate-to-severe atopic dermatitis with dupilumab and concomitant topical corticosteroids (LIBERTY AD CHRONOS): a 1-year, randomised, double-blinded, placebo-controlled, phase 3 trial. *The Lancet*.

[6] Rabe K. F., Nair P., Brusselle G. (2018). Efficacy and safety of dupilumab in glucocorticoid-dependent severe asthma. *New England Journal of Medicine*.

[7] Castro M., Corren J., Pavord I. D. (2018). Dupilumab efficacy and safety in moderate-to-severe uncontrolled asthma. *New England Journal of Medicine*.

[8] Wang C., Kraus C. N., Patel K. G., Ganesan A. K., Grando S. A. (2020). Real-world experience of dupilumab treatment for atopic dermatitis in adults: a retrospective analysis of patients’ records. *International Journal of Dermatology*.

[9] Cork M. J., Thaçi D., Eichenfield L. F. (2020). Dupilumab in adolescents with uncontrolled moderate-to-severe atopic dermatitis: results from a phase IIa open-label trial and subsequent phase III open-label extension. *British Journal of Dermatology*.

[10] Bachert C., Han J. K., Desrosiers M. (2019). Efficacy and safety of dupilumab in patients with severe chronic rhinosinusitis with nasal polyps (LIBERTY NP SINUS-24 and LIBERTY NP SINUS-52): results from two multicentre, randomised, double-blind, placebo-controlled, parallel-group phase 3 trials. *The Lancet*.

[11] Alexis A. F., Rendon M., Silverberg J. I. (2019). Efficacy of dupilumab in different racial subgroups of adults with moderate-to-severe atopic dermatitis in three randomized, placebo-controlled phase 3 trials. *Journal of Drugs in Dermatology*.

[12] Faiz S., Giovannelli J., Podevin C. (2019). Effectiveness and safety of dupilumab for the treatment of atopic dermatitis in a real-life French multicenter adult cohort. *Journal of the American Academy of Dermatology*.

[13] Wenzel S., Castro M., Corren J. (2016). Dupilumab efficacy and safety in adults with uncontrolled persistent asthma despite use of medium-to-high-dose inhaled corticosteroids plus a long-acting *β*_2_ agonist: a randomised double-blind placebo-controlled pivotal phase 2b dose-ranging trial. *The Lancet*.

[14] Fulkerson P. C., Rothenberg M. E. (2013). Targeting eosinophils in allergy, inflammation, and beyond. *Nature Reviews Drug Discovery*.

[15] Xiong X. F., Zhu M., Wu H. X., Fan L. L., Cheng D. Y. (2019). Efficacy and safety of dupilumab for the treatment of uncontrolled asthma: a meta-analysis of randomized clinical trials. *Respiratory Research*.

[16] Ou Z., Chen C., Chen A., Yang Y., Zhou W. (2018). Adverse events of dupilumab in adults with moderate-to-severe atopic dermatitis: a meta-analysis. *International Immunopharmacology*.

[17] Safa G., Paumier V. (2019). Psoriasis induced by dupilumab therapy. *Clinical and Experimental Dermatology*.

[18] Salgüero-Fernández I., Gonzalez de Domingo M. A., Suarez D., Roustan-Gullón G. (2019). Dermatitis and alopecia in a patient treated with dupilumab: a new adverse effect?. *Clinical and Experimental Dermatology*.

[19] Barroso-García B., Rial M., Molina A., Sastre J. (2018). Alopecia areata in severe atopic dermatitis treated with dupilumab. *Journal of Investigational Allergology and Clinical Immunology*.

[20] Menzella F., Montanari G., Patricelli G. (2019). A case of chronic eosinophilic pneumonia in a patient treated with dupilumab. *Therapeutics and Clinical Risk Management*.

[21] Kinoshita Y., Oouchi S., Fujisawa T. (2019). Eosinophilic gastrointestinal diseases–pathogenesis, diagnosis, and treatment. *Allergology International*.

